# Modeling Avoidance in Mood and Anxiety Disorders Using Reinforcement Learning

**DOI:** 10.1016/j.biopsych.2017.01.017

**Published:** 2017-10-01

**Authors:** Anahit Mkrtchian, Jessica Aylward, Peter Dayan, Jonathan P. Roiser, Oliver J. Robinson

**Affiliations:** a Institute of Cognitive Neuroscience, University College London, London, United Kingdom; b Gatsby Computational Neuroscience Unit, University College London, London, United Kingdom

**Keywords:** Anxiety, Avoidance, Diathesis–stress, Pavlovian bias, Reinforcement learning, Threat of shock

## Abstract

**Background:**

Serious and debilitating symptoms of anxiety are the most common mental health problem worldwide, accounting for around 5% of all adult years lived with disability in the developed world. Avoidance behavior—avoiding social situations for fear of embarrassment, for instance—is a core feature of such anxiety. However, as for many other psychiatric symptoms the biological mechanisms underlying avoidance remain unclear.

**Methods:**

Reinforcement learning models provide formal and testable characterizations of the mechanisms of decision making; here, we examine avoidance in these terms. A total of 101 healthy participants and individuals with mood and anxiety disorders completed an approach-avoidance go/no-go task under stress induced by threat of unpredictable shock.

**Results:**

We show an increased reliance in the mood and anxiety group on a parameter of our reinforcement learning model that characterizes a prepotent (pavlovian) bias to withhold responding in the face of negative outcomes. This was particularly the case when the mood and anxiety group was under stress.

**Conclusions:**

This formal description of avoidance within the reinforcement learning framework provides a new means of linking clinical symptoms with biophysically plausible models of neural circuitry and, as such, takes us closer to a mechanistic understanding of mood and anxiety disorders.

Avoidance is a core feature of anxiety (1, 2) and plays a central role in psychological strategies for the treatment of anxiety (3), but its underlying neural and cognitive mechanisms are unknown. Avoidance can be adaptive: if an individual perceives a situation as stressful then it makes sense to avoid that stressor in the future. However, excessive avoidance can result in a pathological downward spiral. The more one avoids a situation, the less opportunity there is to learn that the situation is not as bad as feared, and a vicious cycle of avoidance and impaired extinction learning emerges, which in turn promotes further anxiety (1). For example, an individual who fears social embarrassment might ultimately end up housebound, avoiding all social interaction.

The diathesis-stress model of mood and anxiety disorders (4) proposes that maladaptive avoidance should be greatest during periods of environmental stress in vulnerable individuals. This idea has clear face validity and is supported by clinical anecdotes but is largely derived from retrospective, subjective self-reports. This is because quantifying avoidance under stress in an experimentally controlled yet ecologically valid manner in humans is methodologically challenging. In this study we address this challenge using 1) a translationally validated [i.e., comparable behavioral responses can be elicited across human and animal models (5)] threat-of-shock procedure to induce stress (6, 7); 2) a cognitive task that has been shown to reliably index avoidance behavior in healthy individuals (1); and 3) a computationally precise method of defining of avoidance.

Specifically, we operationalize avoidance as a behavioral bias toward withholding action (no-go [i.e., inhibition]) in the face of potentially negative outcomes. This powerful prepotent reflexive (or pavlovian) bias has been observed consistently in humans and animals (8, 9, 10, 11) and is so profound that it can disrupt instrumental goal-directed behavior (8, 9, 10, 11). This is known as pavlovian-instrumental transfer (12), and we harness it here to measure the degree to which individuals rely on their prepotent avoidance biases. Given that both induced stress (13, 14) and pathological anxiety have been associated with increased inhibitory control, it seems plausible that a combination of stress and anxiety will increase reliance on pavlovian inhibitory avoidance biases (15) [in contrast with depression alone, which might plausibly be associated with reduced reliance on pavlovian approach biases (16)].

Reinforcement learning algorithms can provide parameterizations of avoidance behavior that offer insight into both optimal behavior when set correctly (17) and to dysfunction and pathology when set incorrectly (18). Critically, reinforcement learning models enable us to parameterize the influence of pavlovian avoidance biases on task performance in a formal manner. A large body of work has applied these models to healthy humans (8, 9, 10) and they form the basis of human-level artificial intelligence (17), but to date they have not been applied to individuals with mood and anxiety disorders.

We therefore tested individuals with mood and anxiety disorders and healthy individuals completing an approach-avoidance go/no-go task under stress, which was induced by threat of shock. Avoidance was defined and parameterized within a reinforcement learning framework. We predicted that the mood and anxiety group would show high reliance on avoidance bias and that this avoidance bias would be exacerbated by stress.

## Methods and Materials

### Participants

All data, task scripts, and code to recreate the figures in this article are freely available online (https://figshare.com/articles/Avoidance_Anxiety_Materials/3860250). A total of 101 participants were included in the study. Healthy participants (*n =* 58 [originally *n =* 62 but 4 individuals were excluded because they failed to follow task instructions]; 36 men [62.1%]; age range = 18–57 years; mean ± SD age = 26.7 ± 7.1 years) and unmedicated individuals with pathological mood and anxiety symptoms (*n =* 43; 27 men [62.8%]; age range = 18–53 years; mean ± SD age = 28.8 ± 8.8 years) were recruited from online advertising and institutional subject databases. The primary difference between the groups in initial recruitment was that only the pathological group self-defined as experiencing distress from mood/anxiety symptoms. We recruited a mixed sample of anxiety and depression diagnoses because they are highly comorbid with overlapping symptoms and may not therefore represent truly distinct pathologies. Healthy participants responded to an advertisement asking for healthy individuals with no psychiatric symptoms. A phone screen confirmed no history of psychiatric, neurological, or substance use disorders. The mood and anxiety group responded to an advertisement for individuals suffering from low mood, anxious, or depressive symptoms. Following an initial phone screen, individuals who met criteria for mood or anxiety disorder symptomatology according to a face-to-face Mini-International Neuropsychiatric Interview (19) were included. According to the Mini-International Neuropsychiatric Interview, the majority of participants (*n =* 27) met criteria for both generalized anxiety disorder and major depressive disorder (MDD) (*n =* 9 with additional panic disorder), generalized anxiety disorder (*n* = 8; *n =* 3 with panic disorder, *n =* 1 with agoraphobia), panic disorder and MDD (*n* = 2), and MDD alone (*n* = 6; Supplemental Table S1). The average number of depressive episodes was 5 ± 7. The average duration of episodes was 7 ± 8 months (excluding one participant who reported a continuous episode since adolescence). Further details are provided in the Supplement.

### Manipulation

State anxiety was induced via threat of unpredictable electric shocks delivered with two electrodes attached to the nondominant wrist using a Digitimer DS5 Constant Current Stimulator (Digitimer Ltd., Welwyn Garden City, United Kingdom). A highly unpleasant (but not painful) subjective shock level was established using a shock work-up procedure prior to testing. No more than five (to avoid habituation) shocks with gradual increasing shock level were administered. Participants rated each shock on a scale from 1 (barely felt) to 5 (unbearable). Shock level was matched at a level of four across participants. The experimental task was programmed in Psychtoolbox-3 (http://psychtoolbox.org) for MATLAB R2014b (version 8.4.0.1) (The MathWorks, Inc., Natick, MA), presented on a laptop and administered under alternating safe and threat blocks. During the safe block, the background color was blue and preceded by a 4000-ms message stating, “You are now safe from shock.” During the threat block, the background color was red and the message stating “Warning! You are now at risk of shock” was presented for 4000 ms. Participants were told that they might receive a shock only during the threat condition but that the shocks were not dependent on their performance. In practice, a single shock was delivered at a pseudorandom time point during one third of threat blocks (a total of four shocks across 480 trials). Note that it is the anticipation of these shocks, not the shocks themselves, that constitutes the manipulation (see the Supplement). At the end of each experimental task, participants retrospectively rated how anxious they felt during the safe and threat conditions on a 10-point Likert-type scale with responses ranging from 1 (not at all) to 10 (very much so).

### Approach-Avoidance Task

**Figure 1:**
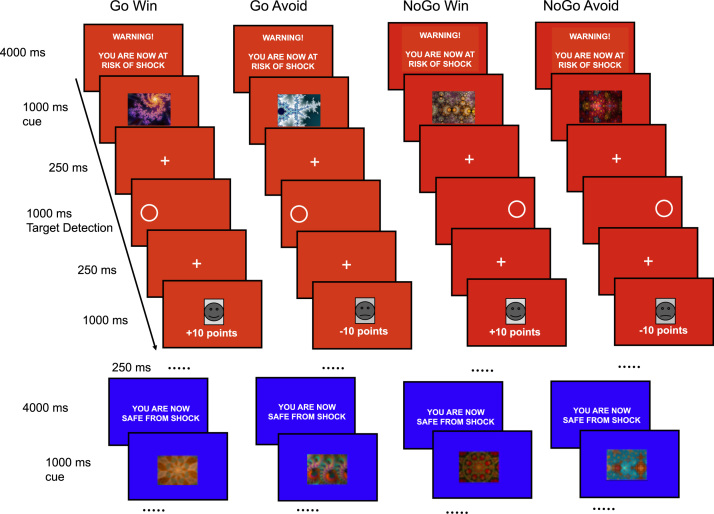
Experimental paradigm. The trial sequence for each trial-type condition under threat (red) and safe (blue) conditions. There were equal numbers of go to win, go to avoid, no-go to win reward, and no-go to avoid losing trials within each safe and threat block, and these were randomly ordered within each block (note that safe sequence proceeds in the same way as the threat sequence but is curtailed here for brevity). The prepotent pavlovian bias to a win is a go response (approach) and the prepotent pavlovian response to a loss is no-go (avoid); hence in go to win reward and no-go to avoid losing, the bias and task instructions are aligned, but in go to avoid losing and no-go to win reward participants have to learn to overcome their avoidance and approach biases, respectively. The safe and threat blocks were presented in alternating order, counterbalanced across participants. A different set of fractal cues was used for the safe and threat blocks, counterbalanced across participants. At feedback, a face (happy +10 points, fear –10 points) was shown 80% of the time, and no points (i.e., a yellow bar [not shown in the figure]) was shown 20% of the time.

The task was based on the design of a previous probabilistic go/no-go reinforcement learning task (10, 20) modified to incorporate the threat manipulation. The prepotent pavlovian bias to a win is a go response (approach), and the prepotent pavlovian response to a loss is a no-go (avoid) response. As such, the task comprised four experimental conditions where action (go/no-go) was crossed with valence (reward/punishment): 1) go to win reward, 2) go to avoid losing (GA), 3) no-go to win reward (NGW), and 4) no-go to avoid losing. On each trial, participants were presented with one of four fractal cues per condition, followed by a target detection task and subsequently by a probabilistic outcome (Figure 1; more task detail in the Supplement).

### Reinforcement Learning Models

Reinforcement learning modeling proceeded in the same way as described in a prior article (10). Briefly, we built seven parameterized reinforcement learning models to fit to the behavior of the subjects. All models were adapted Rescorla-Wagner models. We use the term “standard” to denote the six-parameter winning model from Guitart-Masip *et al.* (10) and either add or subtract parameters to test model fits for seven separate models (see Table 1 for a parameter specification summary).

**Table 1:** Model Specification

Model Name	NP	Parameter							
Standard – Action Bias	5	Reward sensitivity	Punishment sensitivity	Learning rate		Lapse	—	Approach-avoid bias	
Standard – Approach-Avoid	5	Reward sensitivity	Punishment sensitivity	Learning rate		Lapse	General action bias	—	
Standard + 2 Approach-Avoid – 1 Sense	6	Sensitivity		Learning rate		Lapse	General action bias	Approach bias	Avoidance bias
Standard	6	Reward sensitivity	Punishment sensitivity	Learning rate		Lapse	General action bias	Approach-avoid bias	
Standard + 2 Approach-Avoid	7	Reward sensitivity	Punishment sensitivity	Learning rate		Lapse	General action bias	Approach bias	Avoidance bias
Standard + 2 Learning Rates	7	Reward sensitivity	Punishment sensitivity	Reward learning rate	Punishment learning rate	Lapse	General action bias	Approach-avoid bias	
Standard + 2 Approach-Avoid + 2 Learning Rates	8	Reward sensitivity	Punishment sensitivity	Reward learning rate	Punishment learning rate	Lapse	General action bias	Approach bias	Avoidance bias

NP, number of parameters.

#### Learning Models

All the models assigned a probability to each action *a*_*t*_ on trial *t* based on an action weight and the current stimulus. The action weights were constructed according to a simple Rescorla-Wagner–like update equation with a learning rate. Reinforcements were coded as +1 for a reward, –1 for a punishment, and 0 for no feedback. A sensitivity parameter determined the effective size of reinforcements for a subject. For the majority of models the sensitivity parameter could take on different values for the reward and punishment trials. For one model (standard + 2 approach-avoid – 1 sense) there was only one sensitivity parameter per subject, thus assuming that failure to obtain a reward was as aversive as obtaining a punishment. The initial value for the go action was set to zero, and the action weight was modified to include a static general action bias parameter, which denoted overall go tendency (with the exception of one model [standard – action bias] in which this was not included). The pavlovian approach-avoid bias parameter (excluded for one model [standard – approach-avoid]) inhibited the tendency to go in proportion to the negative value of the punishment stimulus, while it similarly promoted the tendency to go in proportion to the positive value of the reward stimulus. For the model with two approach-avoid parameters (standard + 2 approach-avoid), there were two parameters, updated separately for rewarded and punished trials. For the models with two learning rates (standard + 2 approach-avoid + 2 learning rates or standard + 2 learning rates), there were separate learning rates for rewarded and punished trials. In sum, for a given action (*a* = go or no-go), stimulus (*s* = go to win reward, GA, NGW, or no-go to avoid losing), or reinforcement (*r* = +1, –1, or 0) on each trial *t*:

(1) $$Q_{t\left(a_{t},s_{t}\right)}=Q_{t-1\left(a_{t},s_{t}\right)}+LearningRate\cdot \left(\left(Sensitivity\cdot r_{t}\right)-Q_{t-1\left(a_{t},s_{t}\right)}\right)$$

(2) $$Value_{t\left(s_{t}\right)}=Value_{t-1\left(s_{t}\right)}+LearningRate\cdot \left(\left(Sensitivity\cdot r_{t}\right)-Value_{t-1\left(s_{t}\right)}\right)$$

(3) $$ActionWeight_{t\left(a,s\right)}=\{\begin{array}{cc} Q_{t\left(a,s\right)}+ActionBias+AppAvoBias\cdot Value_{t(s)} & a=go\\ Q_{t\left(a,s\right)} & a=nogo \end{array}$$

#### Observation Model

For action selection, the probability of each action was passed through a squashed softmax function with the addition of an irreducible lapse parameter (referred to as “noise” in earlier papers, but renamed “lapse” here to avoid confusion with temperature noise parameters), which was free to vary between zero and one.

(4) $$ActionProbability_{\left(a_{t},s_{t}\right)}=\;\left[\frac{\exp(ActionWeight_{t(a_{t},s_{t})})}{\sum_{a\prime }\exp(ActionWeight_{t\left(a\prime ,s_{t}\right)})}\right]\cdot \;\left(1-Lapse\right)+\frac{Lapse}{2}$$

### Parameter Estimation

We used a hierarchical type II maximum likelihood expectation–maximization procedure to fit the parameters across all subjects and conditions. These procedures are identical to those used by Huys *et al.* (12). Briefly, the top level of the hierarchical model specified distributions over the parameters for the subjects (see below). At each iteration, the current top-level distributions were used as a prior for a Laplace approximation to the intermediate-level posterior distribution of the parameters for each subject (the E-phase). These intermediate-level distributions were then used to determine the next iteration of the top-level distributions (the M-phase). The algorithm was initialized with maximum likelihood values of all the parameters for the subjects; the Laplace approximation was based on the use of fminunc in MATLAB, using multiple random initial values at each iteration of optimization to help avoid local minima. Four different population distributions were tested:

1. Four distributions: one for anxious individuals under threat, one for controls under threat, one for anxious individuals under safe, one for controls under safe. This is the most relaxed procedure and serves to pull all parameters apart.
2. Two distributions: one distribution for threat and one distribution for safe. This fitting procedure was blind to the existence of group.
3. A single distribution for all participants and conditions (i.e., each participant was included twice within the distribution; once for the safe condition and once for the threat condition). This fitting procedure was blind to the existence of both group and threat condition, and serves to pull all parameters closer together.
4. Two distributions: one distribution for anxious individuals and one distribution for control subjects. This fitting procedure was blind to the existence of induced anxiety.

The fit of each model and distribution was compared using the integrated Bayesian information criterion (iBIC). The iBIC is the integral of the likelihood function over the individual parameters [for details, see (12)]. Small iBIC values indicate a model that fits the data better after penalizing for the number of parameters. The parameter fitting procedure results in one iBIC per distribution. These are then summed together to provide a single iBIC to enable model comparison across distributions. The lowest overall iBIC denotes the winning model and distribution combination [an approximate Bayes factor of the comparison of iBIC scores can be calculated using exp(ΔiBIC/2).] Note that fitting the parameters of the winning model using a different hierarchical Bayesian approach recovered similar parameters (see the Supplement). During fitting, parameters are constrained to within meaningful ranges [see (12)]. Exponential transforms are applied to ensure that approach-avoid and sensitivity parameters do not go below zero and sigmoid transform to ensure that learning rate and action bias parameters are constrained between zero and one. These transformations mean that parameters are not normally distributed.

The parameters recovered from the winning model were then compared across groups and conditions using two-tailed permutation tests implemented R coin (https://cran.r-project.org/web/packages/coin/index.html; IndependenceTest, oneway_test). The recovered *p* values are comparable to those derived from standard *t* tests, but do not require the assumption of normality (critical given the possibility of multimodal distributions recovered from the model fitting procedure).

## Results

### Basic Analysis of Symptoms and Behavior

**Figure 2:**
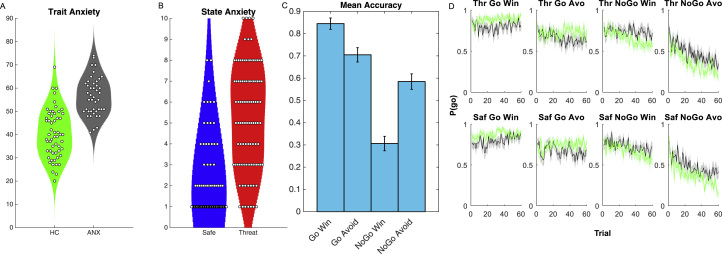
Self-report anxiety and task performance. Between groups, **(A)** our mood and anxiety sample reported significantly higher trait anxiety scores (data missing for two participants in the control group [HC] [green] and one in the mood and anxiety group [ANX] [gray]), while **(B)** the whole sample reported increased (induced) anxiety, rated retrospectively, under threat relative to safe (Saf) conditions (violin plots; each point represents a subject, background shading represents estimated distribution). **(C)** Collapsed mean accuracy differs as a function of trial type, but this ignores that **(D)** performance on the task changed over time, such that the probability of making a response [P(go); as distinct from accuracy in panel **(C)**] differed as a function of trial type, condition, group, and time (shading represents SEM). Avo, avoid; Thr, threat.

As expected, the mood and anxiety group reported significantly higher symptoms of trait anxiety (*F*_1,96_ = 69.6, η_p_^2^ = .4, *p <* .001; Figure 2A) and depressive symptoms (*F*_1,90_ = 50, η_p_^2^ = .4, *p <* .001) relative to controls (for a breakdown by subdiagnosis, see Supplemental Table S1; note that as is commonly observed these measures are highly correlated across the whole sample [*r*_96_ = .755, *p <* .001]). Participants retrospectively reported feeling greater anxiety during the stress manipulation relative to the matched safe condition (*F*_1,99_ = 166, η_p_^2^ = .6, *p <* .001; Figure 2B), which was similar between groups (main effect of group [*F*_1,99_ = 2.0, η_p_^2^ = .02, *p =* .16]; group × condition interaction [*F*_1,99_ = 0.007, η_p_^2^ < .001, *p =* .9]).

Analysis of overall performance accuracy revealed a main effect of action (*F*_1,99_ = 90, η_p_^2^ = .5, *p <* .001), qualified by an action (go/no-go)-by-valence (reward/punishment) interaction (*F*_1,99_ = 94, η_p_^2^ = .5, *p <* .001; Figure 2C). As expected, this was driven by worse relative performance in the conditions where pavlovian biases had to be overcome in order to make the appropriate response (i.e., a loss-driven avoidance bias in GA and a win-driven approach bias in NGW) as well as an overall bias toward making go responses (which means that no-go performance is worse overall likely due to subjects’ prior belief that they should respond). There was a main effect of group (*F*_1,100_ = 15, η_p_^2^ = .1, *p <* .001) driven by worse overall accuracy in the mood and anxiety group, but there were no other interactions with group or condition (all *p* values >.5). However, as apparent in Figure 2D, learning follows a complex time course that differs by condition (and by individual). We therefore turned to a computational model-based analysis to integrate the results across conditions, and thereby examine these differences at a fine scale. In the Supplement, we exploit this clearer understanding to show model-agnostic signatures of the model-based effects.

### Reinforcement Learning Model Selection and Validation

**Figure 3:**
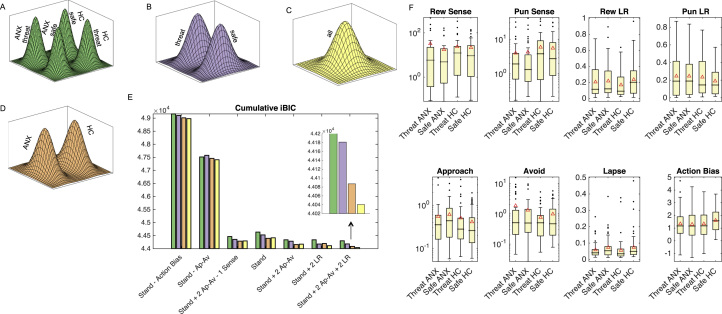
Model fitting and comparison. Four different population distributions were tested separated by **(A)** group and threat condition (four distributions); **(B)** by threat condition alone (two distributions); **(C)** blind to group and threat condition (one distribution); and **(D)** by group alone (two distributions). Comparison of models and distributions using integrated Bayesian information criteria (iBIC) scores (colors match distributions throughout figure) revealed a winning model of standard + 2 approach-avoid + 2 learning rates, fit across a single prior distribution (inset zoomed in on the distribution comparison for this model). Box-and-whisker plots of the recovered parameters from the wining model/distribution are presented in panel **(F)** separated by group and condition (red triangles denote means, lines denote medians; based on individual parameter estimates). Log scales are used for the sensitivity and approach-avoidance parameters to aid visualization of these exponentially transformed parameters. ANX, mood and anxiety group; Ap-Av, approach avoid; Approach, approach bias; Avoid, avoidance bias; HC, healthy control group; LR, learning rate; Pun, punishment; Rew, reward; Sense, sensitivity; Stand, standard.

We fitted reinforcement learning models to trial-by-trial choice behavior using an hierarchical type II maximum likelihood expectation–maximization approach (12). The most parsimonious model (standard + 2 approach-avoid + 2 learning rates; Table 1; Figure 3E; Methods and Materials) is an adapted Rescorla-Wagner model (21) identical to the winning model in prior studies of healthy individuals (8, 10), with the exception that there are separate pavlovian approach, avoid, and learning rate parameters for the cases of rewards and punishments. In other words, this model included an approach bias parameter, an avoidance bias parameter, and accommodated separate speeds of learning about rewards and punishments.

The hierarchical model fitting procedure requires the specification of population-level priors. This raises an important conceptual question when it comes to considering multiple groups. Should we consider mood and anxiety and healthy groups as being sampled from the same or different populations? We answered this question through the adoption of a population-level model comparison approach. We compared fits for models ranging from four separate prior distributions for each group and stress condition (Figure 3A) to a single distribution for all subjects and conditions (Figure 3C). The best fit for our winning model was achieved by fitting a single population distribution (Figure 3C), implying that we did not obtain sufficient evidence to suggest that anxious and healthy individuals were sampled from different populations. Box plots and means of the posterior parameter distribution across subjects (under the type II maximum likelihood expectation–maximization approach) are shown in Figure 3F; that all subjects share the same prior implies that the recovered parameters will be drawn closer together.

**Figure 4:**
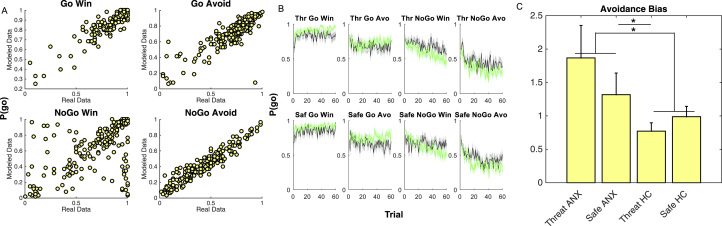
Posterior predictive model. Running the estimated parameters for each subject through a posterior predictive model recovered both **(A)** average go probabilities for each trial type (sensitivity plots: each marker represents one subject under one condition so there are twice as many markers as subjects) and **(B)** group-averaged trial-by-trial performance (compare to real data in Figure 2C). In panel **(B)** green shows healthy control group (HC) and gray shows mood and anxiety group (ANX). Comparing parameters across group and condition revealed **(C)** a significantly higher avoidance bias parameter in pathological anxiety across conditions as well as greater threat-potentiated avoidance in pathological anxiety (error bars represent SEM). Avo, avoid; Saf, safe; Thr, threat.

We next ran a posterior predictive model with parameters set to those from the winning model (i.e., having a computer make decisions as if it were each individual subject). Average parameters recovered from simulated data were close to those that were originally observed (Figure 4A), albeit with more noise for the NGW condition. Average simulated behavior over time matched closely that of the subjects (Figure 4B; compare to Figure 2C; see also Supplemental Table S2).

### Pathological Symptoms Are Associated With Increased Reliance on Avoidance Bias, Especially Under Stress

We finally performed permutation tests on the posterior parameters to assess the effects of group and threat condition. These revealed an increased reliance on the avoidance bias parameter in the mood and anxiety group (effect of group averaged across threat and safe: *p*_permutation_ = .042; Figure 4C) and a significantly greater increase in the avoidance parameter under the threat condition versus the safe condition in the mood and anxiety group relative to control subjects (*p*_permutation_ = .015; Figure 4C) driven by a significantly greater avoidance in the mood and anxiety group relative to control subjects under the threat condition (*p*_permutation_ = .006) but not the safe condition (*p*_permutation_ = .17) (there was no significant condition effect within groups [mood and anxiety group *p*_permutation_ = .36; control subjects *p*_permutation_ = .28]).

## Discussion

Anxious individuals show strong avoidance behavior that can be debilitating and self-perpetuating (1, 2). Here, using a computational approach, we provide evidence that mood and anxiety disorders are associated with increased reliance on an avoidance bias (a pavlovian bias to withhold responding in the face of punishments) during reinforcement learning. Moreover, consistent with the diathesis-stress hypothesis, this effect was exacerbated under stressful conditions in the mood and anxiety group only.

We provide a potential computational mechanism for this effect. We show that avoidance behavior—which is currently measured by retrospective self-report—can emerge at the level of stimulus-action associations. Specifically, individuals with mood and anxiety disorders may show avoidance in the face of threats because they inhibit their action tendencies when faced with a perceived negative outcome. This is consistent with prior work demonstrating increased behavioral inhibition under stress (13, 14), in pathological anxiety (15), and in high (nonpathological) trait anxiety (22) [although see (23)]. Over time, however, individuals may be ultimately able to learn to overcome this bias (i.e., promote instrumental override of pavlovian bias parameters) if they are given the opportunity to experience outcomes (i.e., NGW go probability is lower at the end than go to win reward here). However, in the real world, avoidance means that, by definition, predicted outcomes are rarely experienced and challenged, there is little opportunity to learn, and a persistent miscalibration can emerge.

The growing field of computational psychiatry (18) seeks to use theory-driven approaches to explain psychiatric phenomena. Testable theories are a prerequisite to a clear mechanistic understanding: here, we have outlined a precise and formalized computational theory about how avoidance emerges in anxiety under stress. This approach has at least two further advantages. First, it allows us to reduce a highly dimensional dataset (here, choices over time) into a small number of parameters that respect the temporal variability of the data (unlike responses averaged over time). Second, we can directly integrate this model into biophysically plausible models of underlying neural activity (24). Indeed, performance of this task in healthy individuals has been linked neurocognitively to striatal and midbrain regions associated with network models of action (9, 10) as well as dopaminergic modulation of this circuitry (25). Striatal regions of this circuitry are also modulated by the threat of shock technique used here (26), providing a link between these substrates and stress. This computational approach therefore holds promise as a means of unifying complex psychiatric phenomena, such as avoidance, with their underlying neural circuitry.

Such a mechanistic link is critical if we wish to develop improved treatments. Without mechanistic understanding, treatment development has to be targeted at downstream symptoms (e.g., self-reported avoidance). The problem with this approach can be illustrated by the symptom of cough (27). Lung cancer, allergies, bronchitis, or tuberculosis all result in a cough through fundamentally different mechanisms, but the treatment for one will be ineffective for the others (and indeed may even cause harm through side effects). Targeting clearly defined mechanisms, not symptoms, should ultimately improve the effectiveness of interventions. For example, extensive work in the development of psychological interventions for mood and anxiety disorders has suggested that exposure therapy should be paired with behavioral training to overcome avoidance to be effective (1), but the mechanism is unclear. The present findings suggest that this may be because such training encourages an instrumental override of pavlovian bias during action selection. One avenue for future exploration, therefore, is whether training to overcome bias on GA trials on tasks such as in the present study could promote instrumental override [cf. (28) but also (29)]. If proven effective, such speculative task-based interventions (completed via smartphones, for example) could have enormous potential value for public health.

### Limitations

While our model may provide a mechanism by which avoidance behavior occurs in anxiety and depression, it does not provide a means of disentangling its relationship with specific constructs under the broad category of distress (30). Indeed, symptoms of anxiety and depression are highly comorbid (mixed MDD and generalized anxiety disorder is the most common diagnosis in our sample and our self-report measures of anxiety and depression are highly correlated), so future work is needed to delineate how, if at all, avoidance processes map separately onto feelings of anxiety or depression. In this study we did not find a reliable relationship between the avoidance parameter and self-reported anxiety symptoms using a dimensional approach (see the Supplement). One potential explanation is that our self-report measures are not optimal for capturing the symptoms measured by our task. Self-reported avoidance behavior might, for instance, show a stronger relationship with task performance.

It is also worth highlighting that there is a difference between passive avoidance and active avoidance, the latter being where an individual performs an action to avoid harm (i.e., GA). There are clear individual differences in avoidance learning strategies (31), so reliance on active versus passive avoidance may differ across subgroups of anxious individuals. For instance, active avoidance may be especially prominent in posttraumatic stress disorder (32), so an interesting question for future work is whether posttraumatic stress disorder may be associated with corresponding improved GA performance and hence improved task performance.

Another important limitation is that while it is possible to see evidence of the influence of the avoidance parameter when performance averages are divided into separate time bins (see Supplement), our nonmodeling analysis is inherently less sensitive to the avoidance effects because focusing on means reduces our sensitivity to detect effects that evolve over trials.

Finally, it should be noted that we use a Bayesian framework for evaluating model fit and then use a frequentist approach to compare output parameters. This approach asks whether parameters, which were fitted under a single distribution, actually come from separate distributions. This is highly conservative and will require large effects in order for differences to be detected. A better approach would be to test the effect of varying the population priors at the parameter level. In light of the present data, we would predict that avoidance bias would be best fit using multiple distributions, while all other parameters will be best fit under a single distribution. This would enable inference about group differences in parameters to be fully confined within the model comparison framework. We are actively developing tools that will enable this approach in the future. Relatedly, this is the first study using this task to report results for a model that includes separate avoidance and approach parameters. To the best of our knowledge this model has not previously been reported, and it is possible that it would also offer the most parsimonious account of other samples. However, it is also plausible that the addition of an extra parameter is only warranted in a sample in which this captures additional variance (as is the case here, being the only parameter that differs across groups).
