# Supplementary material for: Modeling Avoidance in Mood and Anxiety Disorders Using Reinforcement Learning
Source: Biol Psychiatry. 2017 Oct 1;82(7):532–9. doi: 10.1016/j.biopsych.2017.01.017 (PMC5598542; doi:10.1016/j.biopsych.2017.01.017)
Supplement: Supplementary file 1 — Supplementary material [file mmc1.pdf]

Modeling Avoidance in Mood and Anxiety Disorders Using Reinforcement Learning

Supplemental Information

Additional Participant Information

Exclusion criteria were any form of medication within the last 6 months, any current psychiatric diagnosis (other than major depression or anxiety disorder), neurological or substance use disorders, or pacemaker. Previous unsuccessful treatments greater than 6 months prior to testing included medication (N=5), psychological treatment (N=8) or both medication and psychological treatment (N=12). A small number were undergoing current psychological treatment (N=5) but still met diagnostic criteria and 1 participant with MDD narrowly missed the diagnostic threshold (according to the MINI) but was included due to clear evidence of distress (interaction of interest remains ($p_{permutation}$=0.03) excluding this participant). A measure of trait anxiety was obtained using the State-Trait Anxiety Inventory (STAI; data missing from three participants) (1) and recent depressive symptoms using the Beck Depression Inventory (BDI; data missing for nine participants) (2). All participants provided written informed consent and were reimbursed £7.50/hour for participation. The study obtained ethical approval from the UCL Research Ethics Committee (Project ID Numbers: 1764/001 and 6198/001).

Sample size was determined using *a priori* power calculations. The healthy control sample was powered for an effect size of d=0.49 based on the t-test of the within-subjects effect of an anxiogenic manipulation on a different action valence task (3): N=57 gives 95% power for a two-tailed t-test with α=0.05. The size of the pathological group was based on an assumed between-groups effect size of 1.09 (observed in our prior study: (4)), which was decreased to 0.8 for the purpose of a conservative power analysis. N=42, gives 95% power for a two-tailed between-groups t-test with α = 0.05. Non computational analyses were completed using JASP (5).

Table S1. Self-report symptoms split by sub diagnosis.

Sub Diagnosis	N	Mean STAI (SD)		Mean BDI (SD)	
GAD	8	51	(7)	11	(5)
MDD	6	53	(6)	20	(8)
MDD AND GAD	27	58	(8)	22	(10)
MDD AND PANIC	2	72	(2)	30	(2)

Additional Task Details

The fractal cue, target detection task and the outcome were each presented for 1000ms and separated by a 250ms inter-trial interval (ITI). Each fractal cue signified one of the four experimental conditions, but this was not made explicit at the start of the experiment. Thus, subjects had to learn that each fractal image indicated both which 1) action (go=make response; no-go=withhold response) to perform during the target detection task and 2) the associated valence of the outcome (reward/no reward; punishment/no punishment). The meaning of the fractal cues was randomized across participants. In the target detection task, a circle was presented randomly on one side of the screen (50% of trials on the left). In the go experimental conditions (GW/GA), participants had to match the position of the circle by pressing the corresponding key (i.e., press the left key when the circle was on the left and vice versa). In the no-go experimental conditions (NGW/NGA), participants had to withhold any response (i.e. any response was recorded as incorrect). The circle was presented for 1000ms regardless of response.

In the rewarded conditions (GW/NGW), correct responses were rewarded 80% of the time, but resulted in no win 20% of the time. Incorrect responses led to no win 80% of the time, but were rewarded 20% of the time. In the punishment conditions (GA/NGA), correct responses avoided punishment 80% of the time but led to a loss 20% of the time (and vice versa for incorrect

responses). Wins were indicated by a happy face and a gain of 10 points. Losses were indicated by a fearful face and a 10 point deduction. These were purely hypothetical within the structure of the task (i.e. they did not translate into a financial bonus). A horizontal yellow bar indicated when participants neither won nor lost points. Faces were selected from the Ekman facial set and the genders of the faces were counterbalanced across participants.

Participants were informed about the probabilistic nature of the task but they were not told the action-outcome contingencies for each fractal cue. Instead, they were told that they had to learn the correct response for each fractal cue, which could be either a go response or a no-go response, by trial and error. The task was divided into 24 alternating safe and threat blocks (12 blocks of each) with the order of the safe and threat conditions counterbalanced across participants. A different set of fractal cues was used under threat and safe in order to avoid possible confounding effects from learning under the different conditions. The eight fractal cues for threat and safe (four in each condition) were counterbalanced across participants.

Each block had five trials per experimental condition (GW, GA, NGW, NGA), with a total of 20 trials per block. The trials were randomly presented within each block. There were thus a total of 240 trials (60 trials for each fractal cue) per safe or threat condition. The task lasted around 35 min with a single shock delivered in the third, seventh, tenth and twelfth threat blocks. These shocks were always presented in the ITI between trials (the 4th trial of the 3rd threat block, the 18th trial of the 5th threat block, the 10th trial of the 10th threat block and the 2nd trial of the 12th threat block). Critically, these shocks were presented to maximise manipulation efficacy (6) (see analysis of effect on choice behavior below). Prior to the start of the task, participants completed nine practice trials without the threat manipulation. Each outcome appeared three times and identical black images were used instead of fractal cues in order to familiarise participants with the task without confounding learning of the action-outcome contingencies.

Effect of Shocks

Comparing pooled performance on the five trials before and after the four shocks (i.e., 20 pre- and 20 post-shock trials) revealed no impact of the shock stimulation on accuracy (pre- vs post-shock: $F(1,99)=1.6$, $p=0.21$, $\eta_p^2=0.016$; pre- vs post-shock*group: $F(1,99)=0.7$, $p=0.39$, $\eta_p^2=0.007$) or reaction time (pre- vs post-shock: $F(1,98)=0.13$, $p=0.72$, $\eta_p^2=0.001$; pre- vs post-shock*group: $F(1,98)=0.1$, $p=0.75$, $\eta_p^2=0.001$). An analysis separate by trial type (GA, NGA, GW, NGW) revealed no significant interactions (all $p>0.2$).

Post-hoc Correlational Analysis

If we test the hypothesis that increased mood and anxiety symptoms are associated with increased avoidance we see a weak one-tailed trend towards a relationship with trait anxiety (avoidance parameter under threat $r(99)=0.164$, $p=0.052$ and safe $r(99)=0.146$, $p=0.074$) but nothing in the depression symptoms (threat $r(98)=0.044$, $p=0.335$ and safe $r(98)=0.037$, $p=0.360$). Similarly, if we test for a positive relationship between self-reported impact of the manipulation (i.e. anxiety rating) during the threat condition we find a weak one-tailed relationship with avoidance ($r(101)=0.175$, $p=0.040$), but not approach parameters under threat ($r(101)=0.002$, $p=0.491$).

Model Inspired Basic Analysis

One of the main points of the model is to focus the behavioral data across all conditions precisely onto psychologically-meaningful parameters. However, it is often possible to use the newfound understanding to discern echoes of the same effects in more direct analyses. In our case, the key parametric difference concerned the avoidance bias, which is expected to have a particularly strong negative effect on performance in the GA condition once learning has progressed far enough to arrange for a sufficiently negative value for the state (and thus a sufficiently strong nogo influence). Indeed, focussing on the final two quartiles of trials, there was a time*condition*group interaction on GA accuracy ($F(1,99)=4.5$, $\eta_p^2=0.04$, $p=0.036$).

Specifically, during the third quartile Pavlovian bias means that patients were worse than controls under threat ($t(99)=-2.6$, $p=0.011$) but not safe conditions ($t(99)=-1.5$, $p=0.13$). By the fourth quartile, a performance deficit also emerged in patients under the safe conditions ($t(99)=-2.7$, $p=0.007$; **Figure S1**).

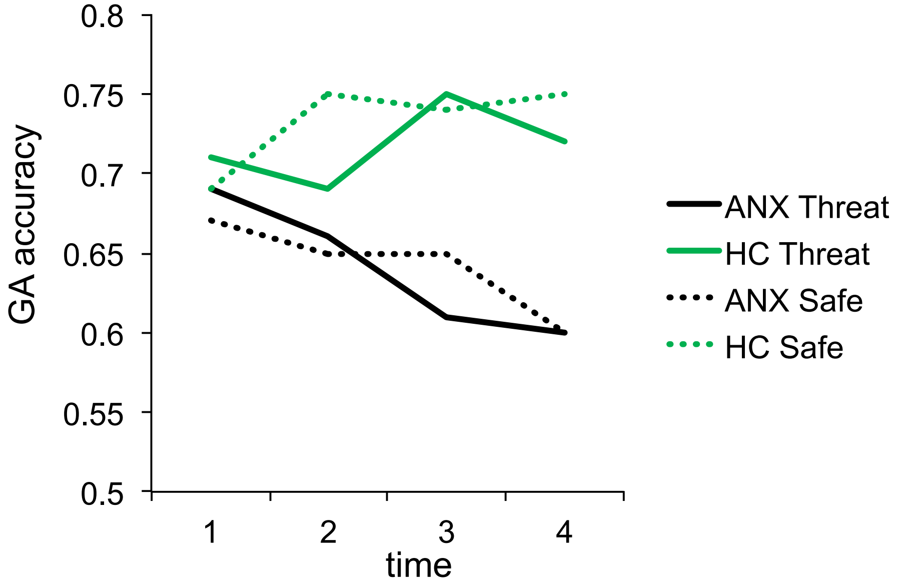

Figure S1. Performance over time (quartile bins) on the go to avoid (GA) trial shows declining performance in the mood and anxiety group as a result of avoidance bias.

Table S2. Behavioral means

**Condition**	**Threat**								**Safe**							
**Action**	**Go**				**NoGo**				**Go**				**NoGo**			
**Valence**	**Win**		**Avoid**		**Win**		**Avoid**		**Win**		**Avoid**		**Win**		**Avoid**	
**Group**	**ANX**	**HC**	**ANX**	**HC**	**ANX**	**HC**	**ANX**	**HC**	**ANX**	**HC**	**ANX**	**HC**	**ANX**	**HC**	**ANX**	**HC**
**Time 1**																
Mean	0.79	0.84	0.69	0.71	0.25	0.26	0.40	0.47	0.75	0.84	0.67	0.69	0.23	0.26	0.39	0.48
SD	(0.18)	(0.19)	(0.20)	(0.19)	(0.23)	(0.26)	(0.25)	(0.25)	(0.21)	(0.18)	(0.20)	(0.20)	(0.24)	(0.25)	(0.23)	(0.19)
**Time 2**																
Mean	0.76	0.87	0.66	0.69	0.27	0.31	0.51	0.63	0.74	0.84	0.65	0.75	0.25	0.29	0.51	0.62
SD	(0.27)	(0.18)	(0.25)	(0.22)	(0.27)	(0.33)	(0.29)	(0.28)	(0.29)	(0.19)	(0.26)	(0.22)	(0.30)	(0.33)	(0.30)	(0.29)
**Time 3**																
Mean	0.78	0.88	0.61	0.75	0.27	0.38	0.58	0.71	0.78	0.90	0.65	0.74	0.31	0.34	0.58	0.66
SD	(0.26)	(0.19)	(0.31)	(0.23)	(0.30)	(0.38)	(0.30)	(0.27)	(0.25)	(0.16)	(0.31)	(0.26)	(0.34)	(0.36)	(0.31)	(0.28)
**Time 4**																
Mean	0.80	0.89	0.60	0.72	0.28	0.42	0.63	0.74	0.83	0.86	0.60	0.75	0.31	0.41	0.60	0.74
SD	(0.26)	(0.20)	(0.33)	(0.27)	(0.33)	(0.42)	(0.32)	(0.26)	(0.25)	(0.19)	(0.30)	(0.24)	(0.36)	(0.39)	(0.34)	(0.25)

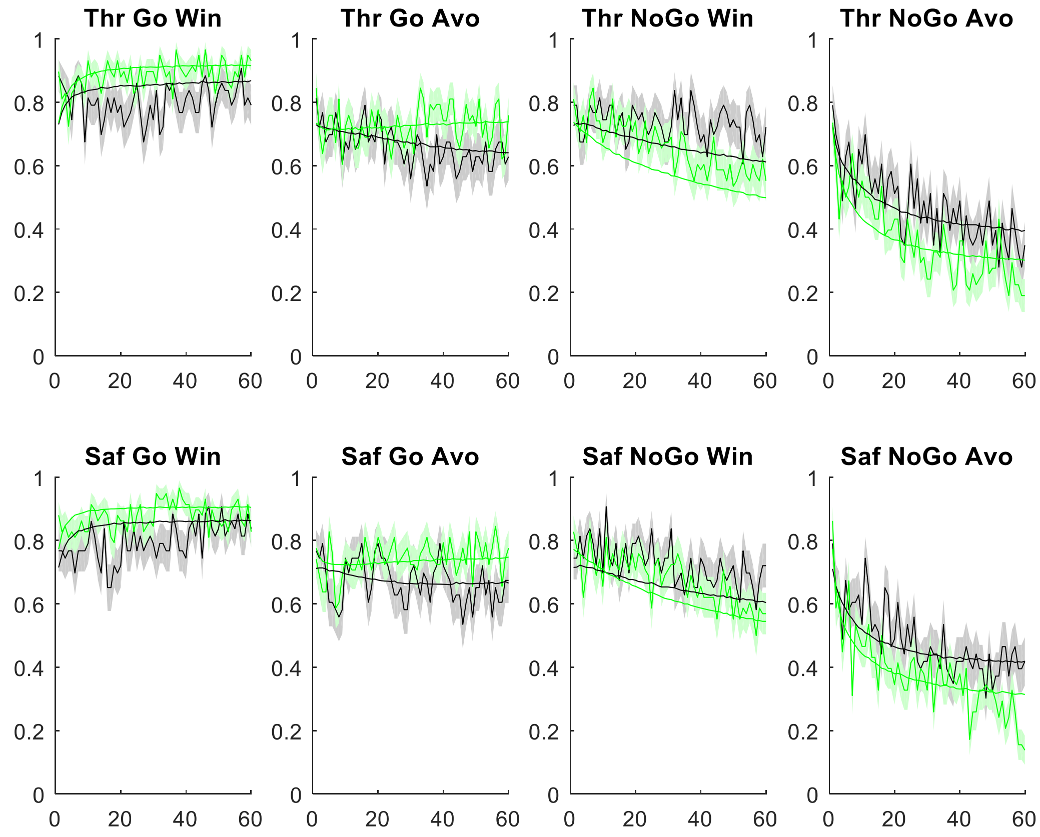

Figure S2. Real data (noisy lines with shaded error bar; replicates figure 2c) overlaid with posterior predictive modelled data (smooth lines) run 1000 times per subject.

Model Fitting

Fitting our winning model using a hierarchical Bayesian approach implemented using the hBayesDM (hierarchical Bayesian modeling of Decision-Making tasks) toolbox (7) recovered very similar avoidance parameters (correlation between parameters: avoidance under threat: r=0.85, p<0.001; avoidance under safe: r=0.84, p<0.001).

We note that our model does a better job of fitting the trials that contribute to the avoidance bias parameter fitting (i.e. the avoid trials; **Figure 4a**) than the rewarded trials (especially NGW). This means that inference is based on the trials that are best captured by the model. Future work might seek to refine model components that improve the fit of the rewarded trials.

Supplementary References

1. Spielberger, C. D., Gorsuch, R. L. & Lushene, R. E. The state-trait anxiety inventory. *Palo Alto, Calif: Consulting Psychologists Press Inc* (1970).
2. Beck, A. T. & Steer, R. A. *BDI, Beck depression inventory: manual*. (Psychological Corporation New York, 1987).
3. Crockett, M. J., Clark, L. & Robbins, T. W. Reconciling the Role of Serotonin in Behavioral Inhibition and Aversion: Acute Tryptophan Depletion Abolishes Punishment-Induced Inhibition in Humans. *The Journal of Neuroscience* 29, 11993-11999 (2009).
4. Robinson, O. J., Cools, R., Carlisi, C. O., Sahakian, B. J. & Drevets, W. C. Ventral striatum response during reward and punishment reversal learning in unmedicated major depressive disorder. *American Journal of Psychiatry* 169, 152-159 (2012).
5. JASP v. 0.5 (2014).
6. Robinson, O. J., Overstreet, C., Charney, D. S., Vytal, K. & Grillon, C. Stress increases aversive prediction-error signal in the ventral striatum. *Proc Natl Acad Sci U S A* (2013).
7. Ahn, W.-Y., Haines, N. & Zhang, L. Revealing neuro-computational mechanisms of reinforcement learning and decision-making with the hBayesDM package. *bioRxiv*, doi:10.1101/064287 (2016).
